# Complementary Feeding Methods—A Review of the Benefits and Risks

**DOI:** 10.3390/ijerph18137165

**Published:** 2021-07-04

**Authors:** Nikki Boswell

**Affiliations:** Queensland Children’s Medical Research Institute (QCMRI), The University of Queensland, Brisbane, QLD 4000, Australia; nikki.boswell@uqconnect.edu.au

**Keywords:** baby-led weaning, complementary feeding, infant feeding

## Abstract

Complementary feeding methods have the potential to not only ensure a diet of nutritional adequacy but also promote optimal food-related behaviours and skills. While the complementary feeding practice known as baby-led weaning (BLW) has gained popularity, evidence supporting the potential benefits and/or risks for infant growth, development, and health warrants consideration. A review of 29 studies was conducted with findings indicating that parents who implement BLW typically have higher levels of education, breastfeed for longer, and differ in other personality traits. Fear of choking was an important factor in parents’ decision not to implement BLW; however, this fear was not supported by the literature. Benefits of BLW included lower food fussiness, higher food enjoyment, lower food responsiveness, and higher satiety responsiveness. While this profile of eating behaviours confers a reduced obesity risk, few studies have examined the relationship between BLW and infant growth robustly. BLW does not seem to increase the risk of inadequate zinc or iron intake; however, emphasis needs to be given to ensuring adequate intake of these micronutrients among all infants. A better understanding of the impacts of BLW is needed to inform evidence-based recommendations to support and guide parents in complementary feeding methods.

## 1. Introduction

During the first year of life, infants progress from an all-milk diet to one that includes nonmilk foods. This transition intends to support the changing nutritional requirements of infants, with implications for long- and short-term growth, development, and health [1]. Given this, the period of transition from a milk-based diet to one that includes solid foods (complementary feeding/weaning) is a critical time in establishing dietary-related practices and behaviours [1]. Ultimately, complementary feeding should lead to the consumption of a variety of foods that meet nutritional requirements, while supporting the acquisition of optimal food-related behaviours, skills, and attitudes. In achieving this, both what and how infants are fed are of importance as integral aspects of an infant’s interpersonal food environment. The interpersonal food environment specifically refers to proximal interactions of the infant with the parents/caregivers, who impose structural boundaries, practices, and norms in relation to food and eating occasions. These interpersonal interactions are significant constructs of the food environment for infants, who rely exclusively on such interactions for the procurement of food and the facilitation of eating occasions [2,3,4,5].

In this regard, while much public health and clinical attention has been given to what infants are fed, as one aspect of the interpersonal food environment, attention to how infants are fed has received less focus. In young children, evidence supports that how children are fed can have an impact on food-related behaviours, such as food fussiness, food responsiveness, and satiety responsiveness, with implications for dietary intake and child weight, growth, and development [6,7,8,9,10]. From this understanding, responsive feeding models have been established as a preferential means of feeding young children, with positive impacts on eating behaviours and dietary intake established across the literature [11,12,13,14,15,16,17]. In responsive feeding, the child leads the feeding interaction by deciding if and how much they will eat, while the parent retains responsibility over what, when, and where a child is fed [14,18]. The parent must refrain from pressuring, coercing, overtly restricting, or rewarding the child with regard to eating [11,12,13,14,15]. This division of responsibility supports a child to self-regulate hunger and appetite, respond appropriately to interoceptive signals, and enjoy a wide variety of nutritious foods [14,18].

Emerging research, largely from the UK and New Zealand, has begun to examine the application of responsive feeding models during the complementary feeding period and the differing impacts on infant growth, development, and health [19,20,21,22,23,24]. Specifically, the responsive complementary feeding practice, known as baby-led weaning (BLW), aims to replicate the division of responsibility whereby parents provide the infant chunks or strips of finger food (deciding what, when, and where the infant is fed) and allow the infant to self-feed, thereby not imposing on how much the infant consumes. With BLW, the infant must engage oral motor skills in a more sophisticated way compared with traditional spoon feeding. That is, the infant is required to manipulate and move chunks of food from the front of the mouth to the back and, consequently, is exposed to the authentic textures and flavours of a variety of foods. Given the unique developmental period of infancy, this method of feeding, while holding much potential to positively shape interpersonal food environments, may also carry inherent risks.

Given the increasing popularity of BLW, and the unique developmental needs of infants, it is important to establish the benefits and/or risks of BLW, as responsive complementary feeding practices, for the growth, development, and health of infants. A better understanding of the impacts of BLW will provide the opportunity to inform evidence-based public health and clinical recommendations, which are necessary to support and guide parents in creating healthful interpersonal food environments during the weaning period. The aim of this review is to develop understanding of the prevalence, practice, and common definitions of BLW, as well as to consolidate evidence of the benefit and/or risk of this responsive feeding method. Discussion will conclude by examining the gaps and limitations in the literature.

## 2. Materials and Methods

### 2.1. Data Source and Search Strategy

A search in the online database PubMed was conducted to identify relevant papers. PubMed was selected as the only search database, which was deemed sufficient for a scoping review and recognized as suitable as a principal search system in a recent evaluation of 28 search databases [25]. Search terms included a combination of the terms “baby-led weaning” OR “baby led” & “weaning” OR “BLW”. Search dates were defined as 2010 until June 2020.

### 2.2. Inclusion and Exclusion Criteria

Original research studies (qualitative and quantitative studies) written in English were included. Studies were included if they focused on the prevalence or processes of BLW and/or outcomes associated with the risks/benefits of BLW to child growth, development, and health. No strict definition of BLW was applied to the included studies; rather, studies self-identified as relating to BLW through the use of the search terms. Studies focusing on the timing of complementary feeding or prolonged milk feeding were excluded. The literature included focused on developed, high-income countries since lower-income countries experience a complexity of issues likely to affect infant feeding practices and infant growth, development, and health that are outside the scope of this review. 

### 2.3. Data Extraction

**Figure 1:**
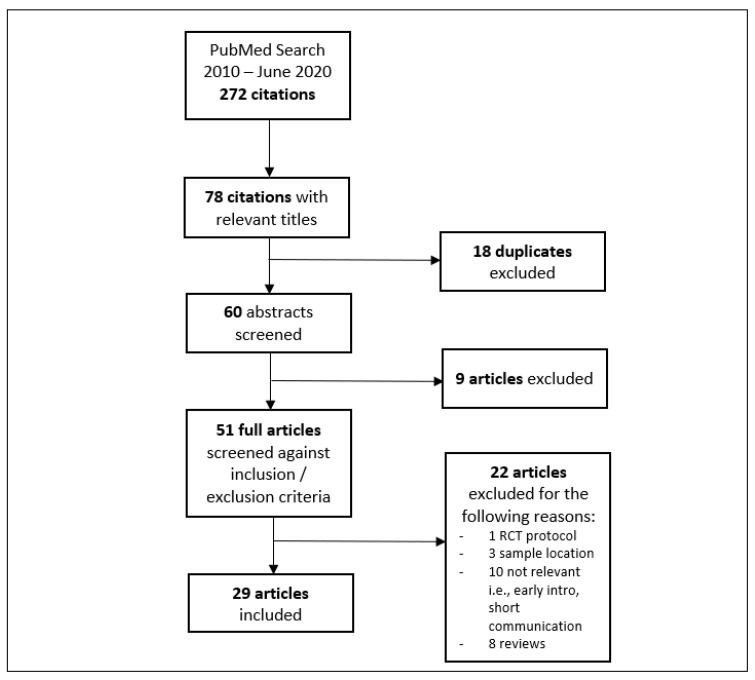
Data extraction process.

Data were extracted for each qualifying paper and recorded on a spreadsheet by a single reviewer. Studies were analysed under categories of focus (i.e., BLW prevalence, eating behaviour outcomes, nutrient intake outcome). Where studies fit more than one category of focus, the dominant focus as indicated by the studies’ primary aim was selected. Figure 1 provides an overview of the article selection process from which data were systematically extracted.

### 2.4. Analysis

A narrative synthesis was performed in order to consolidate the current body of literature and identify gaps in understanding. Studies included in this review were evaluated according to the relevant study quality assessment tools from the National Institutes of Health [26]. These tools comprise a series of questions that assess several potential sources of bias in a study. The areas covered include assessment of measure validity, the suitability of the study design to address research questions, the generalizability of the sample to the population of interest, and the extent to which key confounders are accounted for in the analyses. Based on this, studies were rated as “good”, “fair”, or “poor” [26].

## 3. Results

### 3.1. Findings

A total of 29 articles were identified for inclusion in this review. Five studies were qualitative in nature, while of the quantitative studies, 12 focused on food and nutrient intake; 5 focused on the prevalence, parental experiences, or practical aspects of BLW; 4 focused on eating behaviours (i.e., food fussiness, satiety responsiveness); 3 focused on choking; and no studies were found that focused explicitly on growth (i.e., over- or underweight), although this was also a secondary focus of several studies.

### 3.2. Characteristics of Included Studies

The characteristics of the studies are detailed in Table 1. The studies predominately involved samples from New Zealand, UK, Australia, Canada, or US. Infants were aged from birth to 6.5 years; however, most samples were between 6 and 12 months.

## 4. Discussion

### 4.1. Prevalence, Practice, and Definition of BLW

The popularity of BLW, as a complementary feeding practice, has grown substantially over the past 10 years [24,27]. This increasing popularity has the potential to create a marked shift in the interpersonal environment of infants, with consequences for health, well-being, and development. According to cross-sectional studies from the UK, between 30% and 60% of parents strictly follow BLW practices [27,28]. This rate differs substantially from those reported in New Zealand, wherein only between 8% and 18% of parents indicated they fully implemented BLW practices, while around 70% indicated they followed traditional spoon-feeding methods [23,29]. While sociocultural and population differences between the UK and New Zealand are likely to play a role in explaining these differing rates, so too is lack of a standard definition of BLW practices.

Rapley et al. (2015) broadly defines BLW as “the inclusion of the infant in family mealtimes, where food that is suitable for the infant to eat is made available to all” [30]. More objectively, Brown and Lee (2011b) applied a cutoff of ≤10% pureed food and ≤10% spoon-fed food for a practice to be considered BLW [31]. While many researchers have applied this ≤10% cutoff, a number of other studies simply allow participants to self-define whether they practice BLW or traditional complementary feeding [27,29,32]. Several studies have also been seen to use prompts that describe BLW prior to asking parents to indicate complementary feeding practices, which could induce social desirability bias [27,31]. Further to this, the BLISS RCT specifically classifies adherence to BLW as ≤10% of foods solely parent-fed, ≤15% of foods infant- and parent-fed together, and at least 75% of foods infant-self-fed only [33,34]. The complementary feeding protocol implemented in this trial, however, is considered a modified BLW protocol as parents were given instructions on the implantation of BLW that may differ from authentically implemented practices within free-living populations. That is, parents within the BLISS BLW group were instructed to focus on including high-iron foods (e.g., strips of steak, pate, hummus) and high-energy foods (e.g., avocado, cheese) at each meal, and on reducing the risk of infant choking by avoiding high-risk foods (e.g., raw apple) [33,34]. Importantly, the BLW practices recommended in the BLISS trial were not only considered developmentally appropriate for infants but also considered socioculturally appropriate for the study population (New Zealand). This is likely to be an important consideration with respect to the transferability of the intervention and/or results to other study populations. On this note, it has been reported within free-living populations that while mothers describe BLW in terms of whole foods and self-feeding, in practice mothers indicate also offering purees and infant cereals and assisting the infant in feeding to varying degrees [29,35,36]. This too is important to keep in mind when interpreting data from studies, particularly those in which participants self-defined their participation in BLW practices. 

It is also important to acknowledge that parents who implement BLW practices appear to differ from parents who implement traditional complementary feeding, and thus the impacts and outcomes may not be transferable to different demographic groups. That is, parents who implement BLW typically have higher levels of education, breastfeed for longer, and differ in other personality traits [28,29,31,37,38,39]. Parents who implement BLW practices have also been seen to introduce complementary foods later compared with parents who follow traditional spoon-feeding practices, while adhering to the World Health Organization’s (WHO) recommendation for the introduction of solids at around 6 months of age [28,29,31,37,38,39,40,41]. Many studies also focus on primiparous samples, who may differ from multiparous samples [21,31,42].

In terms of understanding why parents choose different complementary feeding practices, qualitative studies indicate that BLW has ties to ideologies of motherhood and superior parenting [41,43]. This perception of BLW is likely based on media representation of the complementary feeding practices, rather than health-care advice. Across multiple samples, it has been seen that only around 20% of parents indicate they receive information about BLW from health-care professionals, compared with 75%–80% of information from other sources, largely including the Internet and social media [23,36,37]. In line with this, a content analysis of ProQuest International Newsstand (2015) identified 78 news articles that depicted BLW positively, proclaiming it to reduce fussy eating, promote self-regulation, facilitate nutrient-dense food choice by infants, and encourage independence [43]. This is in contrast to perceptions reported by health-care practitioners who had concerns in relation to infant choking and inadequate energy and iron intake [36,44]. In a study by Canadian health-care practitioners (*n* = 33), less than half of those interviewed indicated that they would support BLW within their practice, although these data are somewhat dated [36].

Interestingly, parents who follow traditional “parent-led weaning” appear to share many of the concerns of health-care practitioners, with the main reasons for not wanting to try BLW (as reported by 56% of a New Zealand sample, *n* = 199) being fear of their infant choking (55.3%), concerns about the infant’s ability to eat enough (44.2%), reservation that the infant would not have the necessary motor skills to self-feed (27.6%), or perception that “parent-led feeding” had worked fine previously, so there was no need to change (27.1%) [29]. These concerns appear in contrast to the reasons that mothers chose BLW, as reported in a qualitative study of 13 Australian mothers (46% following BLW) in which “trust” was reported to be a key factor in the decision to follow BLW [41]. That is, trusting their infant to choose foods they could manage and trusting their own instincts, particularly amidst social pressures related to infant feeding [41]. Additional themes identified in this study included “value based versus practical based,” feeding in relation to core values such as fulfilling ideals related to a superior nutritional upbringing for their child versus a desire to be practical in infant feeding [41]. Consistent with this, albeit in contrast to the positive frame of BLW, mothers in this study were inclined to follow traditional feeding (54%) as it was perceived to be an easier and more efficient approach to complementary feeding than BLW, particularly in relation to commercial baby foods in regard to returning to work or other time limitations [41]. This perception of convenience of traditional complementary feeding, interestingly, contradicts the perceptions of health-care practitioners from New Zealand (*n* = 31), who, despite reluctance to recommend BLW, identified convenience as a potential benefit, along with greater opportunity for shared family mealtimes, fewer mealtime battles, healthier eating behaviours, and possible developmental advantages [44]. These are important and noteworthy observations in terms of further analysing the benefits and risks of BLW in order to inform and guide health-care practitioners and provide parents with accurate complementary feeding advice.

### 4.2. BLW: Risks and Benefits

#### 4.2.1. Eating Behaviours

While evidence supports a relationship between parents’ use of responsive feeding practices and healthful eating behaviours in young children, evidence that shows similar benefits of BLW, as a responsive complementary feeding practice, is less well established [6,7,8,9,10,11,12,13,14,15,16,17]. Based on the evidence available, Fu et al. (2018) reported that among 876 infants (6–36 months), those who were fully fed via BLW (defined as “mostly or all self-fed”) had lower levels of food fussiness, as measured using the Children’s Eating Behaviour Questionnaire, compared with those who were traditionally spoon-fed (defined as “mostly or all adult spoon-fed” (difference, 95% CI, −0.37, −0.51 to −0.24) [23]. Similarly, Komninou et al. (2019) showed in a cross-sectional study of 565 infants (12–36 months) that those who were strictly fed following BLW (defined as ‘self-feeding 90% or more of the time’), in comparison with those who were strictly parent-fed (defined as “self-feeding less than 10% of the time”), had significantly lower levels of food fussiness (12.57 ± 0.32 vs. 14.31 ± 0.39, *p* = 0.001), as well as higher levels of food enjoyment (16.39 ± 0.16 vs. 15.84 ± 0.19, *p* = 0.02), after controlling for breastfeeding duration and age of introduction of solid foods [37]. While these findings are consistent with those expected based on studies related to responsive feeding practices in older children, it should be noted that the effect size was small (η2 < 0.03) [6,7,8,9,10,11,12,13,14,15,16,17,37]. Further to this, the opportunity to control for a breadth of responsive feeding/parenting practices (i.e., parental control, encouragement to eat, shared mealtimes) and further demographic variables was missed. This is likely to be an important omission, considering that parents who implemented a strict and predominant BLW style were reported to use less instrumental feeding, less control, and less pressure to eat, while also sharing more mealtimes and eating the same meals more often [37]. As these feeding practices have been seen to be associated with lower levels of obesogenic eating behaviours in older children, it is not possible to determine whether differences in eating behaviours reported can be attributed to BLW specifically or other aspects of responsive feeding [12,45,46,47].

On this note, Brown et al. (2015) similarly showed that at 18–24 months (*n* = 298), infants who were fed via BLW (spoon feeding and purees 10% of the time or less) scored significantly lower on food responsiveness (2.85 (SEM.50) vs. 3.18 (SEM.45), *p* < 0.001) and food fussiness (3.26 (SEM.37) vs. 3.03 (SEM.32), *p* <  0.05), while also scoring higher on satiety responsiveness (2.61 (SEM.43) vs. 2.42 (SEM.38), *p* <  0.05) compared with those following traditional spoon weaning (spoon feeding and purees more than 10%), as would confer an eating behaviour profile associated with a reduced risk of obesity [48,49,50]. Brown et al. (2015) further showed that in the traditional weaning group only, restrictive feeding was associated with lower satiety responsiveness, as an obesogenic eating behaviour trait, while parent concern for infant weight was associated with higher levels of food fussiness at 6–12 months of age [48]. By 18–24 months, however, restrictive feeding was associated with lower satiety responsiveness, while pressure to eat was positively associated with food responsiveness in both weaning groups [48]. These findings are consistent with those expected based on responsive feeding models in older children and further reiterating the importance of controlling for a breadth of responsive feeding practices in order to determine whether suspected differences in eating behaviours can in fact be attributed to infant self-feeding.

Contrary to the potential benefits of BLW on infants’ eating behaviours, as highlighted, Taylor et al. (2017) found that infants following the BLISS BLW protocol had lower levels of satiety responsiveness at 24 months (*n* = 166) compared with those in the control group (adjusted difference, −0.24; 95% CI, −0.41 to −0.07) [34]. Although this eating behaviour trait is considered to confer an increased risk of obesity, no differences were seen in mean (SD) BMI *z*-score at 12 or 24 months [34]. In interpreting this finding, however, it should be remembered that the BLISS RCT was considered a modified BLW protocol, and as such, implemented BLW practices may differ from those implemented in free-living samples. Further to this, and consistent with other studies, responsive feeding practices were also not controlled for in this study, which may alter the results.

#### 4.2.2. Growth (Over- and Underweight)

Consistent with the findings of Taylor et al. (2017), the works of Fu et al. (2018) reported no difference in infant weight-for-age *z*-score at 6–36 months of age based on complementary feeding method; however, these data were obtained by parent report (from their infants’ “Child Well” book) from only 21% of the participants (*n* = 187/876) [23]. Likewise, Alpers et al. (2019) used parent-reported data to conclude that there was no difference in infant (*n* = 134, 6–12 months) weight-for-age centile between BLW (spoon feeding for 10% or less) and traditional weaning (spoon feeding more than 10%) groups [51]. The results reported by Brown et al. (2011a) similarly showed no association between complementary feeding method and infant weight at 6 or 12 months of age (*n* = 604), although these findings were too based on parent-reported infant weight and height [21]. 

A second study by Brown et al. (2015), however, reported that traditionally spoon-fed infants (defined as spoon feeding and purees more than 10%) were significantly heavier than those fed via BLW (defined as spoon feeding and purees 10% of the time or less) at 24 months (F (1, 225) = 7.931, *p* = 0.005) [48]. Further to this, BLW practices were associated with a lower risk of overweight (defined as >85th percentile) at 24 months, although parent-reported anthropometric data were missing for 10% of infants [48]. Consistent with the finding of Brown et al. (2015), albeit concerningly, Townsend et al. (2012) reported that more infants fed via BLW (definition not provided) were classified as underweight (based on BMI *z*-score cutoff of more than −2) compared with traditionally spoon-fed infants (definition not provided; *n* = 3 vs. *n* = 0, respectively) [52]. Conversely, more infants fed via traditional spoon feeding were classified as obese (based on BMI z-score cutoff of more than +2) compared with BLW infants (*n* = 8 vs. *n* = 1, respectively; Fisher’s exact test, *p* = 0.02, two-tailed) [52]. These results should be interpreted cautiously, however, as 32% of BMI data were missing from the BLW group due to being obtained by parent report, while traditional weaning anthropometrics were measured. Further to this, BLW participants were recruited from different sources compared with traditional feeding participants, which could further bias the results [52]. 

As can be seen, few studies have examined the relationship between BLW and infant growth robustly, with the validity of many studies being compromised due to parent-reported infant weight and height data, and much data missing. With this caution in mind, the results lean towards BLW reducing the risk of overweight, and the potential risk of underweight is a serious concern. To better understand the potential risks and/or benefits of BLW in regard to infant growth, more robust studies are needed that include objectively measured infant anthropometrics and longitudinal follow-up. Likewise, with studies on the relationship between BLW and infant eating behaviours, responsive feeding practices and demographic variables should also be controlled for. 

#### 4.2.3. Nutrient Intake 

While the results related to BLW and infant growth appear inconclusive, dietary intake data suggest that infants fed via BLW have no difference in energy intake compared with infants fed via traditional spoon feeding [34,36,51,53,54]. That is, in their analysis of the BLISS RCT, Morison et al. (2018) and Taylor et al. (2017) found no difference in energy intake among infants in the BLW group compared with control based on 3-day weighed food records [34,53]. Likewise, Cameron et al. (2015), who conducted a 12-week BLISS pilot study (*n* = 23), found no difference in energy intake between the weaning groups [54]. While it is important to remember that BLISS is considered a modified BLW protocol, with parents in the experimental condition receiving information and advice regarding what and how to feed their infants, these findings are supported by Morison et al. (2016), who, in a cross-sectional study, found no difference in energy intake between BLW infants (self-identified) and traditionally spoon-fed infants (self-identified) based on weighed food records (1–3 nonconsecutive days, *n* = 51, 6–8 months of age) [36]. Further, consistent with the results of the BLISS RCT, the works of Morison et al. (2016) showed that BLW infants consumed more total fat (48% vs. 42% energy, *p* < 0.001) compared with traditionally weaned infants [36,55]. Alpers et al. (2019) further supported this finding with 24 h dietary recall data from 134 infants (6–12 months of age), where it was reported that BLW infants (defined as spoon-feeding for 10% or less) consumed more fat from food (as distinct from fat from milk) compared with traditionally weaned infants (15.9 g (SD 9.8) vs. 10.2 (SD 8.4), *p* = 0.04) [51]. Across these studies, however, results in relation to saturated fat were inconsistent [36,51,55]. Extending on these findings, Townsend et al. (2012) used a 151-item food preference questionnaire to report that infants (*n* = 155; 20–78 months) fed via BLW (self-identified) had an increased liking for carbohydrates compared with traditionally fed infants (definition not provided), who had preference for sweet foods [52]. While this study should be interpreted cautiously due to recruitment bias, Alpers et al. (2019) also found that at 6–8 months of age, traditionally weaned infants consumed significantly more free sugars than BLW infants (6.5 g (SD 9) vs. 1 g (SD 2.1), *p* = 0.03); however, this difference was not sustained at 9–12 months of age [51].

Moving on from macronutrients, in the previously discussed study by Morison et al. (2016), it was reported that BLW infants consumed less zinc (3.0 vs. 3.7 mg, *p* = 0.001), as well as less iron (1.6 vs. 3.6 mg, *p* < 0.001) and less vitamin B12 (0.2 vs. 0.5 μg, *p* < 0.001), compared with traditionally spoon-fed infants [22,55]. While compromised micronutrient intakes are a particular concern in relation to BLW, no difference in zinc intake was reported by Daniels et al. (2018) in a secondary analysis of the BLISS RCT or by Alpers et al. (2019) in the previously discussed study of 134 infants at 6–12 months [22,51]. Importantly, however, while Alpers et al. (2019) indicated that the mean zinc intake of both weaning groups met the recommended nutrition intake (RNI, UK) of 5 mg, 50% of the BLW infants fell below this RNI [51]. Likewise, iron intake, while not statistically different, was lower than the RNI (7.8 mg) in both weaning groups, with those in the BLW group having substantially lower intake (BLW group, 4.8 mg (SD 2.6) compared with the traditional group (6.2 mg (SD 4.9)) [51]. 

Despite that there was no difference in zinc intake reported in the BLISS RCT, it was reported that infants in the control group obtained most of their zinc from vegetables, compared with the BLW group, who obtained most of their zinc from breads and cereals, at 7 months and 12 months of age [22]. Further to this, it was reported by Alpers et al. (2019) that BLW infants consumed less iron from infant milk (1.6 mg (SD 1.9) vs. 2.4 mg (SD 1.7), *p* = 0.01), while the traditional weaning group was offered significantly more fortified infant cereals (*p* < 0.001) and pre-prepared baby foods (*p* < 0.001), based on results from a food frequency questionnaire (FFQ) [51]. Traditionally weaned infants were also offered more dairy and dairy-based desserts at 9–12 months of age (*p* = 0.04), as well as more salty snacks at 6–8 months of age (*p* = 0.03), compared with the BLW group [51]. Conversely, the BLW group was offered significantly more oily fish (*p* < 0.001) and processed meats (*p* < 0.001) at all ages compared with the traditional weaning group; however, no significant differences were seen for intake of fruits, vegetables, meat and fish, sugary foods, or starchy foods [51].

Consistent with this, Rowan et al. (2019) reported that at 6–8 months of age (*n* = 83), infants fed strictly and loosely via BLW (self-identified from written prompt) had significantly higher exposure to protein than traditionally weaned infants (*p* = 0.002 and *p* = 0.001, respectively) based on a 24 h recall of foods offered to the infant [27]. However, unlike Alpers et al. (2019), the works of Rowan et al. (2019) reported higher exposure to vegetables among infants fed strictly and loosely via BLW (*p* = 0.000 and *p* = 0.016, respectively) [27,51]. In further support of this, in the previously discussed study by Brown et al. (2011b), it was reported that infants fed via BLW (defined as ≤10% pureed food and ≤10% spoon-fed) were most likely to be given fruits or vegetables as first foods (78.9% spoon use and 76.8% puree) compared with traditionally weaned infants, who were mostly given iron-fortified rice cereal as first foods (10.8% spoon use and 14.2% puree use vs. 59.5% spoon use and 62.6% puree use) [31]. These findings are also largely consistent with those of Fu et al. (2018), who reported that, compared with traditionally weaned infants, significantly fewer BLW infants consumed iron-fortified cereals (12% vs. 57%, *p* < 0.001, respectively) at 6 months of age, although they did consume more red meat (68% vs. 52%, *p* < 0.001, respectively) [23]. 

On this note, contrary to other studies, Fu et al. (2018) reported that infants following BLW were less likely to consume fruits and vegetables than traditionally weaned infants when solids were first introduced (8% vs. 19%; *p* = 0.002) [23]. This finding may be of particular importance in consideration of an interesting secondary analysis of the BLISS RCT (*n* = 74, 7–12 months) that aimed to examine differences in infant gut microbiota composition following BLW [56]. In this study, it was seen that traditionally fed infants consumed more “fruits and vegetables” and “dietary fibre” than BLW infants (53 g/day and 1.3 g/day more, respectively), which was suggested to contribute to significantly lower alpha diversity among BLISS BLW infants, compared with traditionally fed infants, at 12 months of age [56]. Mediation models in this study further confirmed that intakes of “fruits and vegetables” and “dietary fibre” explained 29% and 25% of the relationship between the weaning group (BLISS versus control) and alpha diversity, respectively [56]. Although this is the first known study to look into the relationship between weaning method and gut microbiotas, the finding adds a new level of investigation to the risks versus benefits of BLW, with gut microbiota known to have important implications for health and well-being, including changes in eating behaviours, dietary diversity, and body weight [56,57,58,59,60,61]. In interpreting the findings of this study, however, it must be emphasized that findings in relation to vegetable intake and BLW were not consistent across the literature. Likewise, in interpreting the results related to nutritional intake and BLW generally, it should be remembered that many studies were limited due to weaknesses in research methodology, inconsistency in conceptualization and measure of key variables, and lack of control for confounding factors (i.e., parental feeding practices in general, parent eating behaviours, psychosocial variables). With this in mind, the literature reviewed indicates that while there does not seem to be a significant difference in energy intake between BLW infants compared with traditionally weaned infants, some differences in dietary composition appear to exist, particularly in terms of first foods. Although BLW infants do not seem to be at a significantly increased risk of inadequate zinc or iron intake, emphasis needs to be given to ensuring adequate intake of these micronutrients among all infants during the period of weaning.

#### 4.2.4. Choking and Oral Motor Skills

While appropriate food selection during BLW has been discussed thus far in terms of ensuring adequate nutrition, consideration must also be given to appropriate food selection to ensure that the risk of infant choking is minimized. This is a particular concern in relation to BLW due to pieces of food being offered to the infant (rather than purees), who must have appropriate oral motor skills to sufficiently chew the selected food and create a bolus before moving it from the front to the back of the mouth for swallowing. Consequently, the increased chewing skills and stamina required by infants fed via BLW are purported to promote oral motor skills development in comparison with traditionally weaned infants. While no studies were identified that examined differences in oral motor skills development specifically, choking occurrence may offer insight into oral motor risks related to BLW [62]. That is, choking risk may increase if foods included within a BLW regime are not consistent with foods considered developmentally appropriate for an infant’s oral motor skills [62]. In this regard, Özyüksel et al. (2019) retrospectively examined the clinical records of 75 infants (over the past 10 years, 5–12 months) who had undergone bronchoscopy due to foreign body aspiration to show that 80% of aspiration occurred during infant self-feeding, compared with only 14% during caregiver feeding [63]. In this study, the term self-feeding was applied to infants that fully fed themselves and completed a meal without the assistance of a caregiver, which is considered to confer a BLW approach. While these results initially imply a greater choking risk associated with BLW, it is important to note that seeds and nuts were reported to make up the vast majority of foods aspirated by infants in both complementary feeding groups, and as such, it is important that parents and caregivers are aware of the choking risk these foods pose [63]. Further to this, given that Wright et al. (2011) reported that at 6 months of age 40% (*n* = 604) of infants were having some form of finger food (mainly bread, rusks, or biscuits) and by 8 months of age over 90% were having finger foods (but only 51% were having them daily), it is worth noting that in the aforementioned study by Özyüksel et al. (2019), the mean age of aspiration was 9 months of age [40,63].

With this in mind, the literature suggests that infants given finger foods least often have the highest frequency of choking [64,65]. That is, in a study of 1151 infants (4–12 months of age), Brown et al. (2018) indicated that while no difference in choking was seen between weaning groups, among those that had choked (13.6%), infants following a traditional weaning approach (self-identified from written prompt, along with percentage of spoon feeding) experienced significantly more choking episodes for finger foods (F2,147 = 4.417, *p* = 0.014) and lumpy purees (F2,131 = 6.46, *p* = 0.002) compared with infants following a strict or loose BLW approach [64]. Consistent with these results, Fangupo et al. (2016) also reported no significant difference in choking episodes between weaning groups in the BLISS BLW RCT; however, it was reported that infants in the BLISS BLW group gagged more frequently at 6 months of age (relative risk (RR), 1.56; 95% confidence interval (CI), 1.13–2.17) but less frequently at 8 months (RR, 0.60; 95% CI, 0.42–0.87) compared with infants in the control group [65]. This finding, consistent with those of Brown et al. (2018), supports that increased exposure to finger foods through BLW may lead to improved oral motor skills during infancy compared with traditionally weaned infants.

### 4.3. Gaps and Limitations in the Literature

The literature reviewed in this study provides a preliminary picture of the potential risks and benefits of BLW compared with traditionally weaned infants. These findings, however, should be interpreted with caution due to a number of limitations within this field of research. In particular, the fact that there is no standard definition of BLW makes comparison between studies difficult, and thus interpretation of findings should be done so cautiously. Further to this, the dominance of parent-reported infant weight and height in many studies compromises the validity of results related to the relationship between BLW and growth outcomes. Future studies that use clinically recorded measures of growth are specifically needed to address this limitation. Similarly, many studies have failed to control for key variables, particularly parent feeding strategies, breastfeeding, age of commencing complementary feeding, and a range of psychosocial variables, including parent attitudes and motivations related to weaning style. As parents who implement BLW differ substantially in many of these variables, it is important that they are controlled for in order to distinguish any benefits or risks that can be specifically attributed to BLW. It should also be noted that generalizability was a significant limitation in many studies, as was the risk of sample bias due to recruitment methods. Fathers are particularly under-represented in the literature, as are multiparous samples, which could significantly skew data on the experience and practice of BLW. Finally, there is a clear need to conduct longitudinal and cohort studies to extend current findings, which are largely based on cross-sectional studies. Similarly, while there is a need for additional RCT that more closely mimics BLW practices in free-living populations, ethical and practical considerations pose many challenges in achieving this. Caution should also be taken when interpreting the results of this review due to limitations in the inclusion and exclusion criteria applied, which may have introduced bias into the results obtained. That is, since no standard definition of BLW was available, studies were included based on self-identification of BLW protocols or practices, which may have biased the studies included for review. Likewise, the exclusion of studies from lower-income countries may have biased the results obtained, and as such, the results of this study should not be considered transferable outside of high-income countries.

## 5. Conclusions

As can be seen, while there is much promise in relation to the benefits of BLW to infant eating, growth, development, and health, much remains unknown. Based on the evidence available, the literature suggests that BLW may reduce infant food fussiness and increase satiety responsiveness; however, these results are far from conclusive. Likewise, although the current literature hints at BLW reducing the risk of overweight, longitudinal data that control for confounding factors are needed to clarify this. Additionally, while the risk of underweight is a serious concern related to BLW, the literature indicates no difference in energy intake between weaning groups. Similarly, no differences in zinc or iron intake were seen between BLW infants and traditionally weaned infants; however, ensuring adequate intake of these micronutrients among all infants during the period of weaning is important. Likewise, while the risk of choking does not appear to increase among infants following BLW practices and, in fact, BLW may encourage infants to improve their oral motor skills, parents need to be guided to avoid introducing foods that pose a choking risk. On this note and given the increasing popularity of BLW, as a responsive complementary feeding practices, and the unique developmental needs of infants, it is important to expand the current evidence base, such as by better informing parents during the weaning period and developing evidence-based public health recommendations and clinical guidelines.

## Figures and Tables

**Table 1:** BLW study characteristics.

BLW Literature and Study Characteristics							
Reference	N	Study Type	Infant Age Range (Months)	% Parent Respondents Female	Key Focus	Country of Origin	Quality
Qualitative							
McNally (2020)	11	Qualitative study	7–24	100%	Maternal perceptions	UK	Fair
Swanepoel (2018)	13	Qualitative study	NA	100%	Mothers experience	Australia	Fair
Arden (2015)	15	Semistructured interviews	9–15	100%	Mothers experience	UK	Fair
Brown (2013)	36	Semistructured interviews	12–18	100%	Mothers experience	UK	Fair
Cameron (2012)	51	Semistructured interview	NA	NA	Health care professionals’ and mothers’ experiences	NZ	Fair
Prevalence/Parental Experience/Practical							
D’Andrea (2016)	98	Cross-sectional	0–24	100%	Maternal and health care practitioner survey	Canada	Fair
Brown (2016)	604	Cross-sectional	6–12	100%	Maternal characteristics	UK	Fair
Brown (2011a)	604	Cross-Sectional	6–12	100%	Maternal control	UK	Fair
Brown (2011b)	655	Cross-sectional	6–12	100%	Experiences of weaning	UK	Fair
Wright (2011)	604	Prospective cohort	0–12	NA	Gateshead Millennium Study (GMS)	UK	Good
BLW Potential Risks vs. Benefits							
Eating Behaviours							
Komninou (2019)	565	Cross-sectional	12–36	NA	Eating behaviours and feeding style	UK	Fair
Fu (2018)	876	Cross-sectional	6–36	99%	Food fussiness, weight, and choking	NZ	Fair
Taylor (2017)	206	BLISS RCT	7–24	100%	Self-regulation, eating behaviours, energy intake	NZ	Good
Brown (2015)	298	Longitudinal	6–24	100%	Satiety responsiveness and energy self-regulation	UK	Good
Nutrient Intake							
Alpers (2019)	134	Cross-sectional	6–12	NA	Food offered, energy and nutrient intake	UK	Fair
Rowan (2019)	180	Cross-sectional	6–12	99.9%	Dietary composition	UK	Fair
Daniels (2018)	206	Secondary analysis of RCT (BLISS)	7–12	100%	Zinc intakes	NZ	Good
Morison (2018)	206	Secondary analysis of RCT (BLISS)	7–24	100%	Difference in food preference and variety	NZ	Good
Williams Erickson (2018)	206	Secondary analysis of RCT (BLISS)	7–24	100%	Difference in food and nutrient intake	NZ	Good
Daniels (2018)	206	Secondary analysis of RCT (BLISS)	7–12	100%	Zinc status in BLW	NZ	Good
Leong (2018)	74	Secondary analysis of RCT (BLISS)	7–12	100%	Difference in gut microbiota	NZ	Good
Morison (2016)	51	Cross-sectional	6–8	100%	Compare the food and nutrient intake	NZ	Fair/good
Cameron (2015)	23	Pilot intervention	5.5–9	100%	12-week pilot BLISS study	NZ	Fair
Townsend (2012)	155	Case control sample	20–78	Na	Infant feeding and weaning style	UK	Poor
Rowan (2012)	11	2 × cross-sectional	5–6	100%	Parent’s diet, quality of foods offered	USA & UK	Poor/fair
Cameron (2013)	199	Cross-sectional	6–12	100%	Feeding practices and health-related behaviours	NZ	Good
Choking/Oral Motor Development							
Özyüksel (2019)	75	Retrospective study of clinical records	<12	NA	Evaluate foreign body aspiration	Turkey	Good
Brown (2018)	1151	Cross-sectional	4–12	100%	Explored choking frequency	UK	Good
Fangupo (2016)	206	Secondary analysis of RCT (BLISS)	6–12	100%	Explored choking frequency	NZ	Good

## Data Availability

Not applicable.

## References

[1] Schwartz C., Scholtens P.A., Lalanne A., Weenen H., Nicklaus S. (2011). Development of healthy eating habits early in life. Review of recent evidence and selected guidelines. Appetite.

[2] Rosenkranz R.R., Dzewaltowski D. (2008). Model of the home food environment pertaining to childhood obesity. Nutr. Rev..

[3] Brofenbrenner U. (1977). Toward an experimental ecology of human development. Am. Psychol..

[4] Davison K., Birch L.L. (2001). Childhood overweight: A contextual model and recommendations for future research. Obes. Rev..

[5] Story M., Kaphingst K.M., Robinson-O’Brien R., Glanz K. (2008). Creating Healthy Food and Eating Environments: Policy and Environmental Approaches. Annu. Rev. Public Health.

[6] Fildes A., Mallan K.M., Cooke L., van Jaarsveld C.H.M., Llewellyn C.H., Fisher A., Daniels L. (2015). The relationship between appetite and food preferences in British and Australian children. Int. J. Behav. Nutr. Phys. Act..

[7] Albuquerque G., Severo M., Oliveira A. (2017). Early Life Characteristics Associated with Appetite-Related Eating Behaviors in 7-Year-Old Children. J. Pediatrics.

[8] Birch L.L., Doub A.E. (2014). Learning to eat: Birth to age 2 y. Am. J. Clin. Nutr..

[9] Boswell N., Byrne R., Davies P.S.W. (2018). An examination of children’s eating behaviours as mediators of the relationship between parents’ feeding practices and early childhood body mass index z-scores. Obes. Sci. Pract..

[10] Harris H.A., Fildes A., Mallan K.M., Llewellyn C.H. (2016). Maternal feeding practices and fussy eating in toddlerhood: A discordant twin analysis. Int. J. Behav. Nutr. Phys. Act..

[11] Daniels L.A., Magarey A., Battistutta D., Nicholson J.M., Farrell A., Davidson G., Cleghorn G. (2009). The NOURISH randomised control trial: Positive feeding practices and food preferences in early childhood—a primary prevention program for childhood obesity. BMC Public Health.

[12] Daniels L.A., Mallan K.M., Battistutta D., Nicholson J.M., Meedeniya J.E., Bayer J.K., Magarey A. (2014). Child eating behavior outcomes of an early feeding intervention to reduce risk indicators for child obesity: The NOURISH RCT. Obesity.

[13] DiSantis K., Hodges E., Johnson S., Fisher J. (2011). The role of responsive feeding in overweight during infancy and toddlerhood: A systematic review. Int. J. Obes..

[14] Estes P., Anchondo I.M. (2011). Responsive Feeding and Satter’s Feeding Dynamic Models. J. Nutr..

[15] Magarey A., Mauch C., Mallan K., Perry R., Elovaris R., Meedeniya J., Byrne R., Daniels L. (2016). Child dietary and eating behavior outcomes up to 3.5 years after an early feeding intervention: The NOURISH RCT. Obesity.

[16] Hurley K.M., Cross M.B., Hughes S.O. (2011). A systematic review of responsive feeding and child obesity in high-income countries. J. Nutr..

[17] Shloim N., Edelson L.R., Martin N., Hetherington M.M. (2015). Parenting Styles, Feeding Styles, Feeding Practices, and Weight Status in 4–12 Year-Old Children: A Systematic Review of the Literature. Front. Psychol..

[18] Satter E. (1990). The feeding relationship: Problems and interventions. J. Pediatr..

[19] Bell L.K., Jansen E., Mallan K., Magarey A.M., Daniels L. (2018). Poor dietary patterns at 1–5 years of age are related to food neophobia and breastfeeding duration but not age of introduction to solids in a relatively advantaged sample. Eat. Behav..

[20] Blaine R.E., Kachurak A., Davison K.K., Klabunde R., Fisher J.O. (2017). Food parenting and child snacking: A systematic review. Int. J. Behav. Nutr. Phys. Act..

[21] Brown A., Lee M. (2011). Maternal Control of Child Feeding During the Weaning Period: Differences Between Mothers Following a Baby-led or Standard Weaning Approach. Matern. Child Health J..

[22] Daniels L., Taylor R.W., Williams S.M., Gibson R.S., A Fleming E., Wheeler B., Taylor B.J., Haszard J.J., Heath A.-L.M. (2018). Impact of a modified version of baby-led weaning on iron intake and status: A randomised controlled trial. BMJ Open.

[23] Fu X., Conlon C.A., Haszard J.J., Beck K.L., von Hurst P.R., Taylor R.W., Heath A.-L.M. (2018). Food fussiness and early feeding characteristics of infants following Baby-Led Weaning and traditional spoon-feeding in New Zealand: An internet survey. Appetite.

[24] D’Auria E., Pediatrics O.B.O.T.I.S.O., Bergamini M., Staiano A., Banderali G., Pendezza E., Penagini F., Zuccotti G.V., Peroni D.G. (2018). Baby-led weaning: What a systematic review of the literature adds on. Ital. J. Pediatr..

[25] Gusenbauer M., Haddaway N.R. (2020). Which academic search systems are suitable for systematic reviews or meta-analyses? Evaluating retrieval qualities of Google Scholar, PubMed, and 26 other resources. Res. Synth. Methods.

[26] National Institutes of Health Quality Assessment Tool for Observational Cohort and Cross-Sectional Studies. https://www.nhlbi.nih.gov/health-pro/guidelines/in-develop/cardiovascular-risk-reduction/tools/cohort.

[27] Rowan H., Lee M., Brown A. (2018). Differences in dietary composition between infants introduced to complementary foods using Baby-led weaning and traditional spoon feeding. J. Hum. Nutr. Diet..

[28] Brown A. (2016). Differences in eating behaviour, well-being and personality between mothers following baby-led vs. traditional weaning styles. Matern. Child Nutr..

[29] Cameron S.L., Taylor R.W., Heath A.-L.M. (2013). Parent-led or baby-led? Associations between complementary feeding practices and health-related behaviours in a survey of New Zealand families. BMJ Open.

[30] Rapley G., Forste R., Cameron S., Brown A., Wright C. (2015). Baby-Led Weaning:A New Frontier?. ICAN Infant Child Adolesc. Nutr..

[31] Brown A., Lee M. (2011). A descriptive study investigating the use and nature of baby-led weaning in a UK sample of mothers. Matern. Child Nutr..

[32] Brown A., Jones S.W., Rowan H. (2017). Baby-Led Weaning: The Evidence to Date. Curr. Nutr. Rep..

[33] Erickson L.W. (2015). A Baby-Led approach to complementary feeding: Adherence and infant food and nutrient intakes at seven months of age. Doctoral Dissertation.

[34] Taylor R.W., Williams S.M., Fangupo L.J., Wheeler B.J., Taylor B.J., Daniels L., Fleming E.A., McArthur J., Morison B., Erickson L.W. (2017). Effect of a Baby-Led Approach to Complementary Feeding on Infant Growth and Overweight: A Randomized Clinical Trial. JAMA Pediatr..

[35] D’Andrea E., Jenkins K., Mathews M., Roebothan B. (2016). Baby-led Weaning: A Preliminary Investigation. Can. J. Diet. Pr. Res..

[36] Morison B.J., Taylor R.W., Haszard J.J., Schramm C.J., Erickson L.W., Fangupo L.J., A Fleming E., Luciano A., Heath A.-L.M. (2016). How different are baby-led weaning and conventional complementary feeding? A cross-sectional study of infants aged 6–8 months. BMJ Open.

[37] Komninou S., Halford J.C.G., Harrold J.A. (2019). Differences in parental feeding styles and practices and toddler eating behaviour across complementary feeding methods: Managing expectations through consideration of effect size. Appetite.

[38] Brown A., Lee M. (2013). An exploration of experiences of mothers following a baby-led weaning style: Developmental readiness for complementary foods. Matern. Child Nutr..

[39] Arden M.A., Abbott R.L. (2014). Experiences of baby-led weaning: Trust, control and renegotiation. Matern. Child Nutr..

[40] Wright C.M., Cameron K., Tsiaka M., Parkinson K.N. (2011). Is baby-led weaning feasible? When do babies first reach out for and eat finger foods?. Matern. Child Nutr..

[41] Swanepoel L., Henderson J., Maher J. (2020). Mothers’ experiences with complementary feeding: Conventional and baby-led approaches. Nutr. Diet..

[42] McNally J., Hugh-Jones S., Hetherington M.M. (2020). “An invisible map”—Maternal perceptions of hunger, satiation and ‘enough’ in the context of baby led and traditional complementary feeding practices. Appetite.

[43] Locke A. (2015). Agency, ‘good motherhood’ and ‘a load of mush’: Constructions of baby-led weaning in the press. Women’s Stud. Int. Forum.

[44] Cameron S.L., Heath A.-L.M., Taylor R.W. (2012). Healthcare professionals’ and mothers’ knowledge of, attitudes to and experiences with, Baby-Led Weaning: A content analysis study. BMJ Open.

[45] Haycraft E., Karasouli E., Meyer C. (2017). Maternal feeding practices and children’s eating behaviours: A comparison of mothers with healthy weight versus overweight/ obesity. Appetite.

[46] Webber L., Cooke L., Hill C., Wardle J. (2010). Associations between Children’s Appetitive Traits and Maternal Feeding Practices. J. Acad. Nutr. Diet..

[47] Webber L., Cooke L., Hill C., Wardle J. (2010). Child adiposity and maternal feeding practices: A longitudinal analysis. Am. J. Clin. Nutr..

[48] Brown A., Lee M.D. (2015). Early influences on child satiety-responsiveness: The role of weaning style. Pediatr. Obes..

[49] Webber L., Hill C., Saxton J., Van Jaarsveld C.H., Wardle J., Webber L., Hill C., Saxton J., Van Jaarsveld C.H., Wardle J. (2010). Eating behaviour and weight in children. Int. J. Obes..

[50] Boswell N., Byrne R., Davies P.S.W. (2018). Eating behavior traits associated with demographic variables and implications for obesity outcomes in early childhood. Appetite.

[51] Alpers B., Blackwell V., Clegg M.E. (2019). Standard v. baby-led complementary feeding: A comparison of food and nutrient intakes in 6–12-month-old infants in the UK. Public Health Nutr..

[52] Townsend E., Pitchford N.J. (2012). Baby knows best? The impact of weaning style on food preferences and body mass index in early childhood in a case–controlled sample. BMJ Open.

[53] Morison B.J., Heath A.-L.M., Haszard J.J., Hein K., Fleming E.A., Daniels L., Erickson E.W., Fangupo L.J., Wheeler B.J., Taylor B.J. (2018). Impact of a Modified Version of Baby-Led Weaning on Dietary Variety and Food Preferences in Infants. Nutrients.

[54] Cameron S.L., Taylor R.W., Heath A.-L.M. (2015). Development and pilot testing of Baby-Led Introduction to SolidS--a version of Baby-Led Weaning modified to address concerns about iron deficiency, growth faltering and choking. BMC Pediatr..

[55] Erickson L.W., Taylor R.W., Haszard J.J., Fleming E.A., Daniels L., Morison B.J., Leong C., Fangupo L.J., Wheeler B.J., Taylor B.J. (2018). Impact of a Modified Version of Baby-Led Weaning on Infant Food and Nutrient Intakes: The BLISS Randomized Controlled Trial. Nutrients.

[56] Leong C., Haszard J.J., Lawley B., Otal A., Taylor R.W., Szymlek-Gay E.A., Fleming E.A., Daniels L., Fangupo L.J., Tannock G.W. (2018). Mediation Analysis as a Means of Identifying Dietary Components That Differentially Affect the Fecal Microbiota of Infants Weaned by Modified Baby-Led and Traditional Approaches. Appl. Environ. Microbiol..

[57] Tamanai-Shacoori Z., Smida I., Bousarghin L., Loreal O., Meuric V., Fong S.B., Bonnaure-Mallet M., Jolivet-Gougeon A. (2017). *Roseburia* spp.: A marker of health?. Future Microbiol..

[58] Cussotto S., Sandhu K.V., Dinan T.G., Cryan J.F. (2018). The Neuroendocrinology of the Microbiota-Gut-Brain Axis: A Behavioural Perspective. Front. Neuroendocr..

[59] Fetissov S.O. (2017). Role of the gut microbiota in host appetite control: Bacterial growth to animal feeding behaviour. Nat. Rev. Endocrinol..

[60] Lam Y.Y., Maguire S., Palacios T., Caterson I.D. (2017). Are the Gut Bacteria Telling Us to Eat or Not to Eat? Reviewing the Role of Gut Microbiota in the Etiol-ogy, Disease Progression and Treatment of Eating Disorders. Nutrients.

[61] Boswell N., Byrne R., Davies P.S.W. (2018). Aetiology of eating behaviours: A possible mechanism to understand obesity development in early childhood. Neurosci. Biobehav. Rev..

[62] Cichero J.A.Y. (2016). Introducing solid foods using baby-led weaning vs. spoon-feeding: A focus on oral development, nutrient intake and quality of research to bring balance to the debate. Nutr. Bull..

[63] Özyüksel G., Soyer T., Üzümcügil F., Yalçın Ş., Ekinci S., Karnak I., Çiftçi A.Ö., Tanyel F.C. (2019). Foreign Body Aspiration in Infants: Role of Self-Feeding. Pediatr. Allergy Immunol. Pulmonol..

[64] Brown A. (2018). No difference in self-reported frequency of choking between infants introduced to solid foods using a baby-led weaning or traditional spoon-feeding approach. J. Hum. Nutr. Diet..

[65] Fangupo L.J., Heath A.-L.M., Williams S.M., Williams L.W.E., Morison B.J., Fleming E.A., Taylor B.J., Wheeler B.J., Taylor R.W. (2016). A Baby-Led Approach to Eating Solids and Risk of Choking. Pediatrics.

